# Complementary feeding pattern and its determinants among mothers in selected primary health centers in the urban metropolis of Ekiti State, Nigeria

**DOI:** 10.1038/s41598-022-10308-7

**Published:** 2022-04-15

**Authors:** Deborah Tolulope Esan, Oluwaseun Eniola Adegbilero-Iwari, Aishat Hussaini, Aderonke Julienne Adetunji

**Affiliations:** 1 Department of Nursing Science, College of Medicine and Health Sciences, Afe Babalola University, P.M.B. 5454, Ado-Ekiti, Nigeria; 2 Department of Community Medicine, College of Medicine and Health Sciences, Afe Babalola University, Ado-Ekiti, Nigeria; 3 School of Nursing, Lagos University Teaching Hospital, Idi-araba, Lagos, Nigeria

**Keywords:** Medical research, Outcomes research

## Abstract

The incidence of malnutrition in the first two years of life has been directly linked with inappropriate complementary feeding practices along with high infectious disease levels. This study was therefore aimed to assess the complementary feeding pattern among mothers of children aged zero to two years in selected health centres in Ado Ekiti, the capital of Ekiti State, Nigeria. The study was cross-sectional in design. One hundred and thirty-five mothers were selected from two health centres within Ado-Ekiti for this study. A semi-structured interviewer-administered questionnaire was used to collect information from the mothers. The questionnaire included questions that assessed the mothers’ socio-demographic characteristics and complementary feeding pattern. Most (62.5%) infants were introduced to complementary foods at 3–5 months old and water (43.3%) at 3 months of age. The main food item given to the infants on commencement of complementary feeding was formula food (45.9%) followed by fermented cereal gruel (37%). The timing of introduction for different food items revealed that in contrast to the use of fermented cereal gruel (23.8%), fewer children were introduced to iron-rich foods (15.1%) and fruits (11%) at 6 months to a year old. Mother’s knowledge of ideal age for the introduction of complementary feeding (${\chi }^{2}=$ 20.547; p < 0.001) associated significantly with the age of introduction of complementary feeding. More than three-fifth (62.5%) of the respondents had commenced complementary feeding to their infants between 3 and 5 months while an excess of two-fifth (43.3%) of the respondents started giving their children water to drink at 3 months of age. Nurses and nutritionists in primary health care centers should take the lead role in educating mothers about the need for exclusive breastfeeding for the first 6 months of life and appropriate complementary feeding for ages 6–24 months.

## Introduction

It has been established that malnutrition is one of the leading causes of death for many of the world's infants, accounting for over a third of the world's under-five deaths^1–3^. The incidence of malnutrition in the first two years of life has been directly linked with inadequate maternal breastfeeding and complementary feeding practices, along with high infectious disease levels^2,4^.

Complementary feeding is described as the process that starts when breast milk alone is no longer sufficient to meet infants' nutritional requirements, and thus, along with breast milk, other foods and liquids are required^5^. This period is from six months of age to two years and it is the most critical growth period for the child, as nutrition deficiencies may result to chronic long-term health problems such as rickets, iron deficiency anemia, stroke, cancer, coronary heart disease among others^6^.

The food given to children during their weaning period is very crucial as an inadequate complementary diet will significantly inhibit the child's optimum growth, health and cognitive development in the future^7,8^. Consequently, a complementary food should be healthy, adequate and should start timely. However, complementary feeding has been recognized to be one of the most often compromised and wrongly practiced in a child’s developmental stage. Early initiation and improper weaning practices are common practices in cultures around the world^9,10^. While some mothers give their children other diet apart from breast milk right from birth, others delay additional diet until more than nine months, with either case resulting into over or under-nutrition^11^. When complementary feeds are introduced too early (before the age of six months), it may lead to feeding difficulties, increased risk of infection and allergies, early termination of breastfeeding, obesity in children and adults and also affect the feeding behavior of children in the long term^12,13^.

Furthermore, it has been observed that family pressure, cultural beliefs and the environment are some of the factors responsible for inappropriate complementary feeding practices among the Yorubas of South-western Nigeria^14,15^. Other determinants have been attributed to the fact that some mothers lack knowledge on the appropriate diet to give to the growing child and also when to introduce the complementary feeds^16^.

Appropriate feeding practices include the timely introduction of solid and semi-solid foods from six months of age and the enhancement of the quantity and variety of foods consumed by children while maintaining breastfeeding^17^. The World Health Organization (WHO) implemented a number of new metrics in 2008 to serve as the benchmark for evaluating the quality of practices of infant and young child feeding (IYCF)^18^. Based on these metrics, many children in low and middle income countries often do not meet adequate complementary feeding requirements^19^.

Hence, the main objective of this study is to assess the complementary feeding patterns among mothers of children aged 0–2 years in selected primary health centres in Ado Ekiti, Ekiti State, Nigeria. The specific objectives are to:

- Determine the age of introduction of complementary feeding to children in the study.
- Identify the timing of introduction of different food items to the children in the study
- Assess the complementary feeding practices of the mothers in the study setting
- Investigate the factors associated with the age of introduction of complementary feeding in the study.

## Methods

### Study location

The study was conducted in selected basic health centres in Ado-Ekiti, the capital of Ekiti State, Nigeria. Ekiti state is situated entirely within the tropics, in the South-west region of Nigeria. Ado-Ekiti has developed basic infrastructure such as primary schools, secondary schools, private hospitals, three tertiary hospitals and few functional primary health centers. The basic health centers selected for this study; Comprehensive Health Center Oke-Iyinmi (CHCO) and Oke-Oniyo Health Centre Ekute (OHCE) were selected because they are part of the most functional primary health centeres in the State.They are located at the centre of the State capital making them easily accessible to both urban and rural residents. The health centers, are mostly patronised by the indigenous people of Ekiti State, who are predominantly Yorubas, farmers and Civil servants. The health centers render breastfeeding support and baby friendly programmes to mothers usually during both antenatal and post-natal clinics. During the clinics, mothers are educated about infant feeding policy such as importance of breast feeding, usage and risk attached to feeding utensils among others.

### Study population

Inclusion criteria: Mothers of children aged two years and below attending the two selected primary health care centers in Ado Local Government Area, Ekiti State were eligible to participate.

Exclusion criteria: Mothers with infants that were supplemented earlier due to special conditions such as illness and so on, and non-consenting mothers were excluded from the study.

### Study design

The study was cross-sectional in design.

### Sample size

Sample size was determined using Taro Yamane formular^20^:

$$n=\frac{N}{1+N({e)}^{2}}$$

where *n* is the minimum sample size required, *N* is the total population of registered women at both health centers = 178 and $e$ is the sampling error = 0.05. Inputting these values in the above formula, yielded a sample size of 123. In order to compensate for non-response or poorly filled questionnaire, an additional 10% was added to make the sample size 135.

### Sampling procedure

Proportionate stratified sampling technique was adopted to determine the number of respondents from the two health centres (OHCE and CHCO) while random sampling was employed in the selection of participants from each sub-populations. At the time of this study, the proportion of mothers in OHCE (44) to CHCO (133) was 1:3. Hence, the number of respondents recruited from each stratum is calculated as:

Number of respondents selected from OHCE = 1/4 × 135 = 33.8 = 34 participants

Number of respondents selected from CHCO = 3/4 × 135 = 101.3 = 101 participants

A total of 135 participants were recruited for the study based on calculated sample size from the two primary health care centres.

### Data collection instrument

A standardized pretested semi-structured adapted questionnaire was employed for data collection. The adapted questionnaire were from previous studies conducted by^11,21^.

### Validity and reliability of instrument

This study ensured the use of external and content validity. The researchers and two other experts in the field of study closely examined the items in the questionnaire to ensure that they could accurately measure the intended variables. Test–retest method was used to assess the reliability of the instrument. Internal consistency of items showed an intra-class correlation coefficient of 0.75. The questionnaire was administered to 135 respondents at the baby wellness clinic, in the selected primary health care centers in Ado-Ekiti.

### Data management and analysis

The data collected for the study were first of all checked for errors, cleaned and then analyzed using the Statistical Package for Social Sciences (SPSS) version 23.0 for Windows, (IBM Corp., Armonk, N.Y., USA)^22^. Descriptive analysis of socio-demographic characteristics of respondents, age of introduction of complementary food items etc. were presented in frequencies and percentages using tables. The Chi-square test was used to test for significance of association between the independent variables and age of introduction of complementary feeding. P-values < 0.05 were considered statistically significant.

### Ethical consideration

Ethical approval for the study was obtained from the Ethics and Research Committee of Afe Babalola University, Ado-Ekiti. Approval for the study was also obtained from Local Authority of the Primary Health Care board, Ekiti State. Written informed consent was obtained from individual participants before commencement of data collection. In addition, respondents were informed of their right to voluntarily participate or withdraw from the study at any stage without adverse consequences. Confidentiality was also observed as the questionnaire bore no name of respondent or any identifying information. All methods were performed in accordance with the relevant guidelines and regulations.

### Ethics approval and consent to participate

Ethical approval for the study was obtained from the Ethics and Research Committee of Afe Babalola University, Ado-Ekiti.

## Results

A total of 135 mothers were interviewed. Their ages ranged from 17 to 46 years with a mean age of 26.3 ± 4.64 years. Majority (76.3%) of them were aged 20–29 years old. Most of them were married (96.3%), Christians (80%) and of the Yoruba tribe (82.22%). A larger percentage (65.2%) of the participants were self-employed while minority (3.7%) were students. 48.15% of the mothers had secondary education while 8 (5.93%) respondents had no formal education. A higher proportion (35.6%) of the mothers had up to two children while very few (2.1%) had five to eight children. The ages of their children ranged from 1 to 24 months with a mean age of 7.2 $\pm$ 4.39 months and a median age of 6 months. Nearly half (49.6%) of the study children were in the age range of 6–12 months. (Table 1).

**Table 1:** Socio-demographic characteristics of respondents.

Variable	Frequency (n = 135)	Percent (%)
**Age of mother**		
less than 20	5	3.7
20–29	103	76.3
30–39	25	18.5
40 and above	2	1.5
Mean age ± SD	26.3 ± 4.64	
**Marital status**		
Single	5	3.7
Married	130	96.3
**Religion**		
Christianity	108	80.0
Islam	24	17.8
Traditional	1	0.7
Others	2	1.5
**Ethnicity**		
Yoruba	111	82.22
Hausa	8	5.93
Igbo	5	3.70
Others	11	8.15
**Occupation**		
Self-employed	88	65.2
Unemployed	12	8.9
Civil servant	30	22.2
Student	5	3.7
**Level of Education**		
No formal	8	5.93
Primary	3	2.22
Secondary	65	48.15
Tertiary	59	43.70
**Number of Children**		
1–2	91	67.5
3–4	41	30.4
5–8	3	2.1
**Child’s age (months)**		
< 6	53	39.3
6–12	67	49.6
13–19	14	10.4
20–24	1	0.7

Majority (88.9%) of the mothers had introduced their infants to water and other foods. Most (62.5%) infants were introduced to food at 3–5 months old and water (43.3%) at 3 months old. Fewer (2.5%) infants were introduced to food at one month and water at birth. (Table 2).

**Table 2:** Age at introduction of complementary feeding.

Variables	Frequency (n = 120)	Percent (%)
**Child's age when introduced to other foods**		
At day one	5	4.2
At one month	3	2.5
Three-five months	75	62.5
Six months and above	37	30.8
**Child's age when introduced to water**		
At birth	3	2.5
At one month	10	8.3
Three months	52	43.3
Between four and five months	23	19.2
At six months	32	26.7

**Figure 1:**
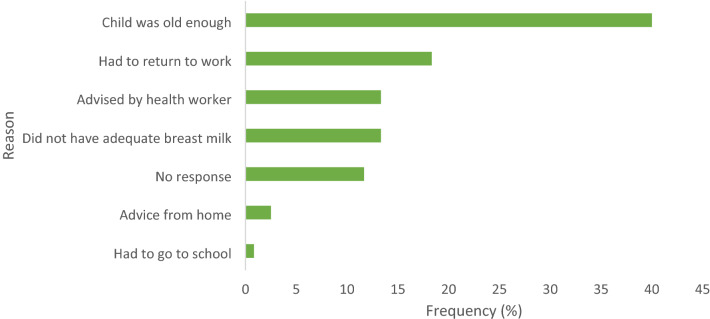
Reason for introduction of complementary food at a particular age.

As shown in Fig. 1, two-fifth (40%) of the study population gave complementary food at a particular age because they felt the child was old enough. The second main reason cited by about 18.33% of the mothers was that they had to return to work after their maternity leave.

Table 3 displays the timing of introduction of some complementary food items by 110 valid respondents. The food items commonly introduced before three months of age were formular food (40%) and skimmed milk (25%). At three to five months, cereals (29.3%) and formular food (26.3%) were prominent. Cereals (23.8%) and animal products such as fish, meat and eggs (15.1%) were more frequently used at six months to a year old. Fruits (29.1%) and vegetables (28.2) were more often introduced to children above a year old.

**Table 3:** Timing of introduction of complementary food items (n = 110).

Variable	< 3 months	3–5 months	6 months-1 yr	> 1 yr	Never given^b^
Cereals^a^	2(10)	49(29.3)	52(23.8)	1(0.9)	6(2.4)
Vegetables	1(5)	5(3)	29(13.3)	31(28.2)	44(17.3)
Fish/Meat/Eggs	0	21(12.6)	33(15.1)	29(26.4)	27(10.6)
Skimmed milk	5(25)	24(14.4)	25(11.5)	5(4.5)	51(20)
Formular food	8(40)	44(26.3)	30(13.8)	3(2.7)	25(9.8)
Cow milk	4(20)	18(10.8)	25(11.5)	9(8.2)	54(21.1)
Fruits	0(0)	6(3.6)	24(11)	32(29.1)	48(18.8)

^a^Including rice, fermented cereal gruel made from maize, millet or guinea corn (pap).

^b^Complementary feeding had not yet been introduced to the infant when mother filled the questionnaire.

When the respondents who had introduced other foods were asked questions on the ideal age for the introduction of complementary feeding, a greater proportion (38.3%) of them felt complementary feeding was appropriate for infants aged 3–5 months old followed by 44 (36.7%) respondents who felt it was appropriate for babies aged 6 months and above (Table 4).

**Table 4:** Complementary feeding practices of mothers.

Variables	Frequency (n = 120)	Percent (%)
**Mother’s knowledge of ideal age for introducing other foods**		
From day one	1	0.8
At one month	2	1.7
Three-five months	46	38.3
Six months and above	44	36.7
No idea	27	22.5
**Still breastfeed with complementary food**		
Yes	103	85.8
**Thickness of child's food**		
Same as other people in the family	3	2.50
Thick enough to stay on a spoon	70	58.33
Watery similar to breast milk	46	38.33
Others	1	0.83
**Feeding a sick child**		
Feed slowly and patiently	87	72.5
Give favorite foods	18	15.0
Feed the child forcefully	15	12.5
**Feeding utensils use**		
Feeding bottle	37	30.8
Bowl and spoon	71	59.2
Hand feeding	7	5.8
Others	5	4.2
**Wash and sterilize feeding utensils**		
Sometimes	18	15.0
Always	101	84.2
Never	1	0.8
**Wash hands before feeding child**		
Sometimes	9	7.5
Always	110	91.7
Never	1	0.8
**Wash hands after changing diaper**		
Sometimes	7	5.83
Always	112	93.33
Never	1	0.83

*Frequency of breast* feeding: Of the 120 (88.9%) mothers who had introduced other foods, a majority (85.8%) breastfed their babies simultaneously with complementary feeding. (Table 4).

*Thickness of child's food and feeding a sick* child: Most of the complementary food given was thick enough to stay on a spoon (58.33%) as the population of the babies comprised mostly of 6–12 month olds (Table 1) followed by watery food texture similar to breast milk (38.33%). While majority (72.5%) of the mothers practiced feeding their babies slowly and patiently in the event of sickness, very few (12.5%) practiced forceful feeding. (Table 4).

*Feeding utensils and Hygiene*: The most commonly used feeding utensil was bowl and spoon (59.2%) followed by feeding bottle (30.8%). About 5.8% still practiced hand feeding which is a norm in the Yoruba culture of south-western Nigeria. Most of the respondents practiced a relatively good hygiene as 84.2% always wash and sterilize feeding utensils, 91.7% wash hands before feeding their children and nearly 93.33% wash hands immediately after changing a diaper. (Table 4).

Majority (80%) of the children breastfed three times or lesser daily, were aged 6 months or older. Surprisingly, a lower proportion (43.5%) of the children breastfed more than thrice daily, were less than 6 months old. These results were comparable but not significant. (Table 5).

**Table 5:** Complementary feeding practices: frequency of breastfeeding child.

	Age of Child			
Frequency of breastfeeding	< 6 months	≥ 6 months	${\chi }^{2}$	p-value
≤ 3 times	1(20)	4(80)	0.334^y^	0.564
> 3 times	50(43.5)	65(56.5)		

${\chi }^{2}$: Chi-square test; ^y^: Yate’s correction.

As presented in Table 6, there were no significant associations between socio-demographic variables such as respondent’s age (p = 0.719), marital status (p = 0.849), religion (p = 0.272), respondent’s ethnicity (p = 0.544), occupation (p = 0.671), level of education (p = 0.636) number of children (p = 1.0) and child’s age (p = 0.108) and age of introduced complementary feeding. However, mother’s knowledge on ideal age for introduction of complementary feeding was significantly associated (*p* < 0.001) with age of introduction of complementary feeding. 91.8% of the mothers who felt complementary feeding was appropriate for infants less than 6 months old introduced their infants to other foods at that period.

**Table 6:** Association between selected variables and age of introduction of complementary feeding.

Variable	Age of introduction			
	< 6 months	$\ge$ 6 months	${\chi }^{2}$	p-value
**Age of mother (years)**				
< 20	4(80)	1(20)	2.329	0.507
20–29	66(71.7)	26(28.3)		
30–39	12(57.1)	9(42.9)		
40 and above	1(50)	1(50)		
**Marital status**				
Single	4(80)	1(20)	0.002^y^	0.964
Married	79(68.7)	36(31.3)		
**Religion**				
Christianity	67(69.8)	29(30.2)	3.214	0.36
Islam	14(66.7)	7(33.3)		
Traditional	0(0)	1(100)		
Others	2(100.0)	0(0)		
**Ethnicity**				
Yoruba	68(68.0)	32(32.0)	2.141	0.544
Hausa	7(87.5)	1(12.5)		
Igbo	2(50.0)	2(50.0)		
Others	6(75.0)	2(25.0)		
**Occupation**				
Self-employed	54(73.0)	20(27.0)	2.713	0.438
Unemployed	8(66.7)	4(33.3)		
Civil servant	19(65.5)	10(34.5)		
Student	2(40.0)	3(60.0)		
**Level of Education**				
No formal	4(57.1)	3(42.9)	3.389	0.335
Primary	2(66.7)	1(33.3)		
Secondary	44(77.2)	13(22.8)		
Tertiary	33(62.3)	20(37.7)		
**Number of Children**				
0–3	72(69.2)	32(30.8)	0.000^y^	1.000
4–8	11(68.8)	5(31.2)		
**Child’s age**				
< 6 months	32(80)	8(20)	2.584^y^	0.108
≥ 6 months	51(69.2)	29(30.8)		
**Mother's knowledge of ideal age**				
< 6 months	45(91.8)	4(8.2)	20.547	< 0.001*
≥ 6 months	25(56.8)	19(43.2)		
Don’t know	13(48.1)	14(51.9)		

${{\varvec{\chi}}}^{2}$: Chi-square test; ^y^: Yates correction; *: p-value < 0.05.

## Discussion

Based on the joint recommendation of the WHO and the United Nations Children’s Fund (UNICEF), the introduction of nutritionally-adequate and safe complementary foods to an infant should be at 6 months^7^. However, results from this study showed that most infants were introduced to complementary feeding (62.5%) as early as 3–5 months old and water (43.3%) at 3 months old. These findings is in congruence with previous studies where complementary feeding was introduced as early as 3 months old^23,24^. A number of reasons were given for early introduction of complementary feeding; topmost among them was that child was old enough (40%). This reason has been highlighted in a previous study by Alzaheb (2016)^25^, but differs from prior studies where mothers felt breastfeeding alone was insufficient for the growth and development of their children^1,26^.

Furthermore, the timing of introduction for different food items was looked into in this study. The results revealed that in contrast to the use of cereals (23.8%) such as fermented cereal gruel which is an important source of energy, fewer children were introduced to iron-rich foods (15.1%) and fruits (11%) at 6 months to a year old. This is also the norm in several West African countries including Ethiopia where complementary foods are based on thin cereal gruel and are low in foods from meat, eggs or fish, especially among low-income groups due to socioeconomic factors, taboos, and ignorance^1,27,28^. Likewise, in Guatemala, most family foods used as complementary foods were considerably short in micronutrients such as calcium, iron, zinc, even when adequate amounts of protein, B vitamins and vitamin C were provided^29^. Interestingly, a wide lag exist between the findings in this study and the practice in a developed country like Netherlands where majority of infants had higher intake of iron-rich foods (51.9%), vegetables (57.4%) and fruits (49.6%) at age six months and above^24^. Moreover, the findings of this study also differ from correct practice developed by the World Health Organisation for infant and young child feeding (IYCF) which recommends the consumption of at least four food groups; at least one animal-source food, at least one vitamin A-rich fruit and vegetable, legumes and nuts, eggs, in addition to a staple food (grain, root or tuber) in a day for children at 6 to 23 months of age^18^.

Complementary feeding practices are directly related to healthy children. In this study, breast-feeding on demand (more than thrice daily) was highly practiced. This practice is also common among the Hausas of North-western Nigeria^30,31^. The most commonly used feeding utensil was bowl and spoon (59.2%), which is similar to other studies in Nigeria^32,33^. Bottle feeding (30.8%) and hand feeding (5.8%) were practiced by few respondents. This is in disparity to what was observed in Sudan where 59.2% mothers fed their children with their hands^34^. Most of the respondents practiced a relatively good hygiene as 84.2% wash and sterilize feeding utensils, 91.7% wash hands before feeding a child and nearly 93.33% wash hands immediately after changing a diaper. This is contrary to previous studies done in Bangladesh, Ethiopia and Tanzania, where majority of the mothers had very poor hygienic practice during complementary feeding^35–37^.

The importance of educating mothers on appropriate complementary feeding practices has been underscored in older studies conducted within and outside Africa^16,26,38–40^. The findings of this study further affirm the fact that mother’s knowledge of the ideal age for the introduction of complementary feeding relates with the age of introduction of complementary feeding. 91.8% of mothers who felt complementary feeding was appropriate for infants less than six months old introduced their infants to other foods at that age. This outcome has been substantiated in earlier studies where most mothers were ignorant of the WHO recommended age for complementary feeding initiation^33,41,41^.

## Conclusion

More than three-fifth (62.5%) of the respondents had commenced complementary feeding to their infants between 3–5 months while more than two-fifth (43.3%) started giving their children water to drink at 3 months of age. Mother’s knowledge of ideal age for introduction of complementary feeding was the major factor for early complementary feeding initiation in this study. These findings imply that intervention strategies such as educating mothers about the need for exclusive breastfeeding for the first 6 months of life and complementary feeding from 6 to 24 months, is mandatory particularly at the grassroots to ensure optimal nutrition for children aged two and below.

## Data Availability

The datasets generated/analysed during the current study are available from the corresponding author on reasonable request.
